# Neural Processing of Calories in Brain Reward Areas Can be Modulated by Reward Sensitivity

**DOI:** 10.3389/fnbeh.2015.00371

**Published:** 2016-01-14

**Authors:** Inge van Rijn, Sanne Griffioen-Roose, Cees de Graaf, Paul A. M. Smeets

**Affiliations:** ^1^Division of Human Nutrition, Wageningen University and Research CentreWageningen, Netherlands; ^2^Image Sciences Institute, University Medical Center UtrechtUtrecht, Netherlands

**Keywords:** brain reward circuitry, calories, maltodextrin, reward sensitivity, sucrose, taste

## Abstract

A food's reward value is dependent on its caloric content. Furthermore, a food's acute reward value also depends on hunger state. The drive to obtain rewards (reward sensitivity), however, differs between individuals. Here, we assessed the association between brain responses to calories in the mouth and trait reward sensitivity in different hunger states. Firstly, we assessed this in data from a functional neuroimaging study (van Rijn et al., 2015), in which participants (*n* = 30) tasted simple solutions of a non-caloric sweetener with or without a non-sweet carbohydrate (maltodextrin) during hunger and satiety. Secondly, we expanded these analyses to regular drinks by assessing the same relationship in data from a study in which soft drinks sweetened with either sucrose or a non-caloric sweetener were administered during hunger (*n* = 18) (Griffioen-Roose et al., 2013). First, taste activation by the non-caloric solution/soft drink was subtracted from that by the caloric solution/soft drink to eliminate sweetness effects and retain activation induced by calories. Subsequently, this difference in taste activation was correlated with reward sensitivity as measured with the BAS drive subscale of the Behavioral Activation System (BAS) questionnaire. When participants were hungry and tasted calories from the simple solution, brain activation in the right ventral striatum (caudate), right amygdala and anterior cingulate cortex (bilaterally) correlated negatively with BAS drive scores. In contrast, when participants were satiated, taste responses correlated positively with BAS drive scores in the left caudate. These results were not replicated for soft drinks. Thus, neural responses to oral calories from maltodextrin were modulated by reward sensitivity in reward-related brain areas. This was not the case for sucrose. This may be due to the direct detection of maltodextrin, but not sucrose in the oral cavity. Also, in a familiar beverage, detection of calories *per se* may be overruled by a conditioned response to its flavor. In conclusion, the brain reward response to calories from a long chain starch sugar (maltodextrin) varies with trait reward sensitivity. The absence of this effect in a familiar beverage warrants further research into its relevance for real life ingestive behavior.

## Introduction

In our Western society, there is an abundance of food cues and an enormous supply of different kinds of appetizing and calorie-rich foods. Therefore, many of us easily engage in overeating. Consequently, it is no surprise that obesity rates are high and still increasing (Ng et al., 2014). However, it is still unclear why some of us are more inclined to engage in overeating than others.

The answer may lie in how sensitive we are to the food rewards surrounding us. Reward sensitivity is a personality trait that can be described as “the ability to derive pleasure or reward from natural reinforcers like food, and from pharmacological rewards like addictive drugs” (Davis et al., 2004). Reward sensitivity can be measured with the Behavioral Inhibition System and Behavioral Activation System (BIS/BAS) questionnaire (Carver and White, 1994). This questionnaire is based on the theory of Gray (Gray, 1990; Carver and White, 1994; Gray and Mcnaughton, 2003), which describes two neurobiological systems that both respond to environmental cues: the Behavioral Inhibition System (BIS) and the Behavioral Approach System (BAS). The BIS is sensitive to signals of punishment, and activation of this system inhibits behavior and induces negative feelings. The BAS is sensitive to signals of reward and activation of this system promotes behavior and positive feelings. Food reward is reflected by the BAS (Carver and White, 1994). More specifically, the BAS is activated by cues that indicate the possibility of attaining food rewards rather than by food consumption (Corr et al., 2013). Sensory signals like taste and sight of food can be seen as such cues, because they signal the presence of nutrients.

High reward sensitivity has been associated with food cravings, overeating, overweight, obesity and eating disorders (Davis et al., 2004, 2007; Franken and Muris, 2005; Bijttebier et al., 2009; Harrison et al., 2010; Verbeken et al., 2012). Beaver et al. (2006) showed that trait reward sensitivity as measured with the BAS scale, is associated with differential processing of food cues in the brain. In their study, reward sensitivity scores of healthy participants correlated strongly with brain activation by pictures of appetizing foods in reward areas such as the ventral striatum, amygdala, midbrain, and orbitofrontal cortex.

Foods are not only rewarding because of their palatability, but also because of their caloric value. Several recent studies found that oral exposure to calories, independent of sweet taste, induced responses in classical reward areas such as the striatum, anterior cingulate cortex (ACC), and amygdala (Frank et al., 2008; Chambers et al., 2009; Smeets et al., 2011) The presence of calories in the oral cavity may directly signal the imminent arrival of a rewarding (caloric) food. Therefore, it is plausible that neural processing of oral calories may be modulated by reward sensitivity in a similar way as was found for food pictures by Beaver et al. (2006). In addition, several studies found that hunger state interacts with brain activation in response to oral calories (Smeets et al., 2011; van Rijn et al., 2015). Currently, though, it is still unknown in how far reward sensitivity differentially affects brain responses to calories during hunger and satiety.

Based on the above, we hypothesized that (1) brain activation in reward areas in response to oral calories depends on trait reward sensitivity, in particular in the striatum, amygdala and ACC, and (2) that this association will be most prominent during hunger. Thus, we aimed to assess the correlation between reward sensitivity and the brain responses to calories in the mouth in different hunger states. Firstly, we assessed this in data from a functional neuroimaging study (van Rijn et al., 2015), in which simple solutions of a non-caloric sweetener with or without a non-sweet carbohydrate (maltodextrin) were administered during hunger and satiety (van Rijn et al., 2015). Secondly, we sought to extrapolate these findings to regular drinks by assessing the same relationship in data from a study in which soft drinks sweetened with either sucrose or a non-caloric sweetener were administered during hunger (Griffioen-Roose et al., 2013). BAS drive and BAS reward, two subscales of the BIS/BAS questionnaire that respectively reflect the tendency to take action in response to a food reward and the amount of positive feelings experienced in response to this reward (Carver and White, 1994; Gomez et al., 2005), were used as measures of reward sensitivity.

## Materials and methods

Data from two separate studies were used. Relevant details are described below. For full experimental details see van Rijn et al. (2015) and Griffioen-Roose et al. (2013).

### Participants

For both studies we recruited healthy, normal-weight (BMI between 18.5 and 25 kg/m^2^) participants (age between 18 and 35 years). Exclusion criteria were among others: a restrained eating score higher than 2.80 (women) or 2.25 (men) (Dutch Eating Behavior Questionnaire (van Strien, 2005), an energy restricted diet during the past 2 months, change in body weight of more than 5 kg during the past 2 months, lack of appetite, stomach or bowel diseases, diabetes, thyroid disease or any other endocrine disorder, use of daily medication other than oral contraceptives, having difficulties with swallowing and/or eating, having taste or smell disorders, being allergic and/or intolerant for products under study, smoking more than one cigarette/cigar a day, exclusive consumption or avoidance of light versions of beverages, being pregnant or lactating or having any contra-indication for MRI scanning. Thirty female participants completed Study 1 and 18 participants completed the fMRI part of Study 2 (15 men, 3 women, see Table 1). Before enrollment, participants were screened on inclusion and exclusion criteria via a questionnaire including a medical history questionnaire and completed an fMRI training session in which they were familiarized with the fMRI procedure. All participants gave written informed consent. Both studies were conducted according to the principles of the Declaration of Helsinki, approved by the Medical Ethical Committee of Wageningen University and registered in the Dutch Trial Register (Study 1: NTR 3749, Study 2: NTR: 3289).

**Table 1:** **Participant characteristics**.

**Characteristics**	**Study 1**	**Study 2**
*N*	30	18
Gender	Female	Male(15)andFemale(3)
BMI (kg/m^2^)^a^	22.6 ± 1.4	22.1 ± 1.6
Age (years)^a^	22 ± 3	22 ± 2
BAS drive score^a^	11 ± 2	12 ± 2
BAS drive range	8–16	9–15
BAS reward score^a^	17 ± 1	18 ± 1
BAS reward range	15–20	15–20

a *Mean ± SD*.

### Study design

Study 1 had a randomized crossover design in which participants were scanned on two occasions, once during hunger and once during satiety. During the two scan sessions participants tasted fixed amounts of a control stimulus (water) and five stimuli containing carbohydrates, artificial sweeteners or both (sucralose, maltodextrin, maltodextrin + sucralose, glucose, and fructose solutions), while their brain responses were measured using functional MRI. Here, we focus on the responses to two of these stimuli, the sweet caloric (maltodextrin + sucralose) and the sweet non-caloric (sucralose) solution.

Study 2 had a randomized crossover design consisting of two periods, which consisted of three parts: a pre-measurement, a conditioning period, and a post-measurement. In the conditioning period, subjects were offered a non-caloric sweetened and sugar sweetened version of a soft drink or a yoghurt drink for breakfast (10 times per drink). During scan sessions in the pre-measurement and post-measurement periods, participants tasted fixed amounts of the non-caloric sweetened and sugar sweetened drinks and a control stimulus (water) while their brain responses were measured using functional MRI. Here, we further analyze the brain responses to tasting the non-caloric sweetened and sugar sweetened soft drinks in the pre-measurement period.

### Stimuli

The sweet non-caloric solution and the sweet caloric solution, used in Study 1, were made by dissolving, sucralose (Brenntag specialties, 0.254 g SPLENDA® Sucralose per liter, 0 kJ/0 kcal per liter) and maltodextrin + sucralose (158.2 g Nutricia Fantomalt (90% polysaccharides − DE 19, 6% mono/disaccharides) + 0.140 g SPLENDA® Sucralose per liter, 2541 kJ/607 kcal per liter) in demineralized water. The solutions were equisweet. Sweetness was matched in a pilot study using the method of constant stimuli (*n* = 10). Furthermore, prior to the study, stimuli were rated on sweetness by a trained sensory panel and during the study by the participants. In both cases, no significant differences in sweetness were found between the two solutions (for more details see, van Rijn et al., 2015).

The non-caloric sweetened and sugar sweetened soft drinks used in Study 2 were developed and prepared by Royal Friesland Campina (Amersfoort, The Netherlands) and contained 0 kJ/0 kcal per liter (0.11 g sucralose per liter) and 1673 kJ/400 kcal per liter (68.6 g sucrose per liter). The soft drinks were grape/lemon flavored and matched on sensory characteristics, including sweetness.

### BAS scores

Reward sensitivity was measured with the Dutch version of the BIS/BAS questionnaire developed by Carver and White (1994). The Dutch BIS/BAS questionnaire was validated by Franken et al. (2005), and is considered a reliable and valid measure. The BAS scale consist of three subscales: BAS drive, BAS reward and BAS fun. BAS drive and BAS reward are most relevant for appetitive motivation and discussed in this paper. “BAS fun reflects the tendency to seek out and impulsively engage in potentially rewarding activities” (Gomez et al., 2005). This scale is not discussed because the food-context of this paper concerns primary reward rather than “activities.” Moreover, we investigate a classic well-known reward (food/calories) rather than a potential reward. In addition, BIS scores are also outside the scope of this paper.

The BIS/BAS questionnaire consists of 20 questions. The BAS drive scale is comprised of four of those questions (min–max score: 4–16) and the BAS reward scale of five (min–max score: 5–20). BAS scores for Study 1 were acquired during the fMRI training session and BAS scores for Study 2 were acquired on the last scan day (after scanning). Scores and ranges of BAS drive and BAS reward for Study 1 and Study 2 can be found in Table 1.

### Experimental procedures

#### Study 1

Participants arrived between 10:25 and 14:00 h at the test location (Hospital Gelderse Vallei, Ede, The Netherlands) after a fast of at least 3 h (no food, only water). Participants were instructed to eat a small self-chosen breakfast, prior to the 3 h fast. Hereafter participants were placed in the MRI scanner and scanned while tasting the solutions several times. During the satiety session participants started with an *ad libitum* lunch consisting of bread rolls (1063 kJ/254 kcal per 100 g), full fat cheese (1570 kJ/375 kcal per 100 g), boiled eggs (645 kJ/154 kcal per 100 g), butter (1549/370 kcal per 100 g), sandwichspread (984 kJ/235 kcal per 100 g), cucumber, tomato, orange juice (167 kJ/40 kcal per 100 g) and skimmed milk (197 kJ/47 kcal per 100 g). Participants were instructed to eat until comfortably full. After lunch, the same procedures were followed as during the hunger session.

#### Study 2

Participants arrived between 7.00 and 11.00 h at the study location (Hospital Gelderse Vallei, Ede, The Netherlands) after a fast of at least 3 h (no food, only water) and were scanned while tasting the soft drinks several times. Note that in this study there was no satiety session.

### Scanning procedure

In study 1, a scan session consisted of a high-resolution T_1_-weighted anatomical scan and 3 functional runs during which 300 functional volumes were acquired using a ${\text{T}}_{2}^{*}$-weighted gradient echoplanar imaging sequence on a 3-T Siemens Magnetom Verio (Siemens, Erlangen, Germany). During each functional run all solutions were tasted four times, resulting in a total of 12 taste trials per solution per scan session. Solutions were offered in 2 mL sips in a semi-random order. Each taste event (11 s) was followed by a 3-s swallow, a 4-s rinse with water, a 3-s swallow and a 3- to 5-s rest.

In study 2, a scan session consisted of a high-resolution T_1_-weighted anatomical scan and 3 functional runs during which 262 functional volumes were acquired using a ${\text{T}}_{2}^{*}$-weighted gradient echo imaging sequence on a 3-Tesla Siemens Magnetom Verio (Siemens, Erlangen, Germany). Each functional run consisted of 5 taste trials for every drink, leading to a total of 15 taste trials per drink. Drinks were offered in 2 mL sips in a semi-random order. Participants tasted every drink for 11 s while a picture of the drink was shown, followed by a 3-s swallow, a 4-s rinse with water, a 3-s swallow and a 3 to 5-s rest.

For both Study 1 and 2, participants rated liking once for every stimulus on a 9-point scale during each functional run. Instructions to either taste, swallow, rate, rinse, or rest were given to participants via visual cues on a screen placed in the bore at the back end of the scanner. Stimuli were administered with the use of programmable syringe pumps (New Era Pump Systems Inc., Wantagh, NY) at 50 mL/min.

### Analysis

In both Study 1 and 2, functional volumes of every participant were preprocessed and analyzed with the SPM8 software package (Wellcome Department of Imaging Neuroscience, London, UK) in conjunction with the MarsBar toolbox (http://marsbar.sourceforge.net/) run with MATLAB 7.12 (The Mathworks Inc, Natick, MA). Details about the preprocessing steps can be found in van Rijn et al. (2015) and Griffioen-Roose et al. (2013).

In the subject level analyses of Study 1, nine conditions were modeled: delivery of sucralose, maltodextrin, maltodextrin + sucralose, glucose, fructose and water, and swallowing, rinsing and stimulus rating. In the subject level analyses of Study 2, seven conditions were modeled: delivery of the non-caloric sweetened soft drink, sugar sweetened soft drink, tomato juice and water, and swallowing, rinsing and stimulus rating. Responses to swallowing, rinsing, stimulus rating, maltodextrin, glucose, fructose, tomato juice and water are not of interest for answering our current research question and are therefore disregarded. After modeling of the conditions, a so-called contrast image was calculated for every participant by subtracting activation by sucralose from activation by maltodextrin + sucralose (Study 1) or activation by the non-caloric sweetened soft drink from that by the sugar sweetened soft drink (Study 2). For Study 1, this was done for both the hunger and satiety condition. Subsequently, these contrast images were entered into separate one-sample *t*-tests with liking, BAS reward and BAS drive as covariates (for Study 1 this was done separate for the hunger and satiety condition). Liking was added as a covariate of no interest to regress out possible effects of differences in liking between the stimuli. Using the other two covariates we tested for correlations between BAS drive/BAS reward scores and taste activation across the whole brain. The resulting correlation T-maps were thresholded at *P* < 0.001 (uncorrected for multiple comparisons) and a cluster size of *k* > 9 contiguous voxels. A priori regions of interest (ROIs) were the amygdala, striatum and ACC. A mask of these regions was created with the WFU Pickatlas tool (Maldjian et al., 2003) and was used to do a ROI-analysis in with small volume correction over the mask volume. Whole brain results are reported in Supplementary Tables 1–3.

## Results

### Main effects

Main effects for study 1 have been reported in van Rijn et al. (2015). There were no differences in taste activation between the maltodextrin + sucrose and sucralose solution. Main effects for study 2 have been reported in Griffioen-Roose et al. (2013). More activation was found for the sugar sweetened soft drink than for the non-caloric sweetened soft drink in the middle cingulum, precentral gyrus and rolandic operculum.

### Correlations between covariates

Pearson correlation coefficients for correlations between the covariates used in the analyses (liking, BAS drive and BAS reward) for Study 1 and 2 can be found in Table 2. BAS drive and BAS reward scores obtained during Study 1 correlated significantly (*r* = 0.38, *P* < 0.05).

**Table 2:** **Pearson correlation coefficients (r) for the correlations between the difference in liking between the caloric and non-caloric stimulus, BAS drive and BAS reward scores**.

**Study**	**Liking and BAS drive**	**Liking and BAS reward**	**BAS drive and BAS reward**
1	H: 0.28	H: 0.05	0.38^*^
	S: −0.08	S: −0.13	
2	−0.03	−0.04	0.28

* *Significant at the 0.05 level; H, hunger; S, satiety*.

### Study 1: sugar solution and reward sensitivity

The ROIs in which correlations between BAS drive scores and brain activation in response to calories (maltodextrin and sucralose minus sucralose) during hunger and satiety were found, are shown in Table 3. BAS drive scores correlated with taste activation in the amygdala, ACC and striatum. BAS reward scores did not correlate with taste activation in any of the ROIs.

**Table 3:** **ROIs in which brain activation by oral calories (maltodextrin and sucralose minus sucralose) correlated significantly with reward sensitivity (BAS drive score) during hunger and satiety**.

**Contrast**	**Region**	**Cluster size**	**Z-score**	**Peak coordinates**		
				***x***	***y***	***z***
**HUNGER**						
Positive correlation	No regions were found					
Negative correlation	R caudate	54	4.15	12	17	−8
	R putamen		4.09	21	17	−8
	R amygdala		3.44	18	11	−14
	R amygdala	21	3.85	18	−1	−17
	R anterior cingulate	74	3.72	3	32	16
	L anterior cingulate		3.33	0	23	22
**SATIETY**						
Positive correlation	L caudate	15	3.76	−12	26	4
Negative correlation	No regions were found					

**Figure 1:**
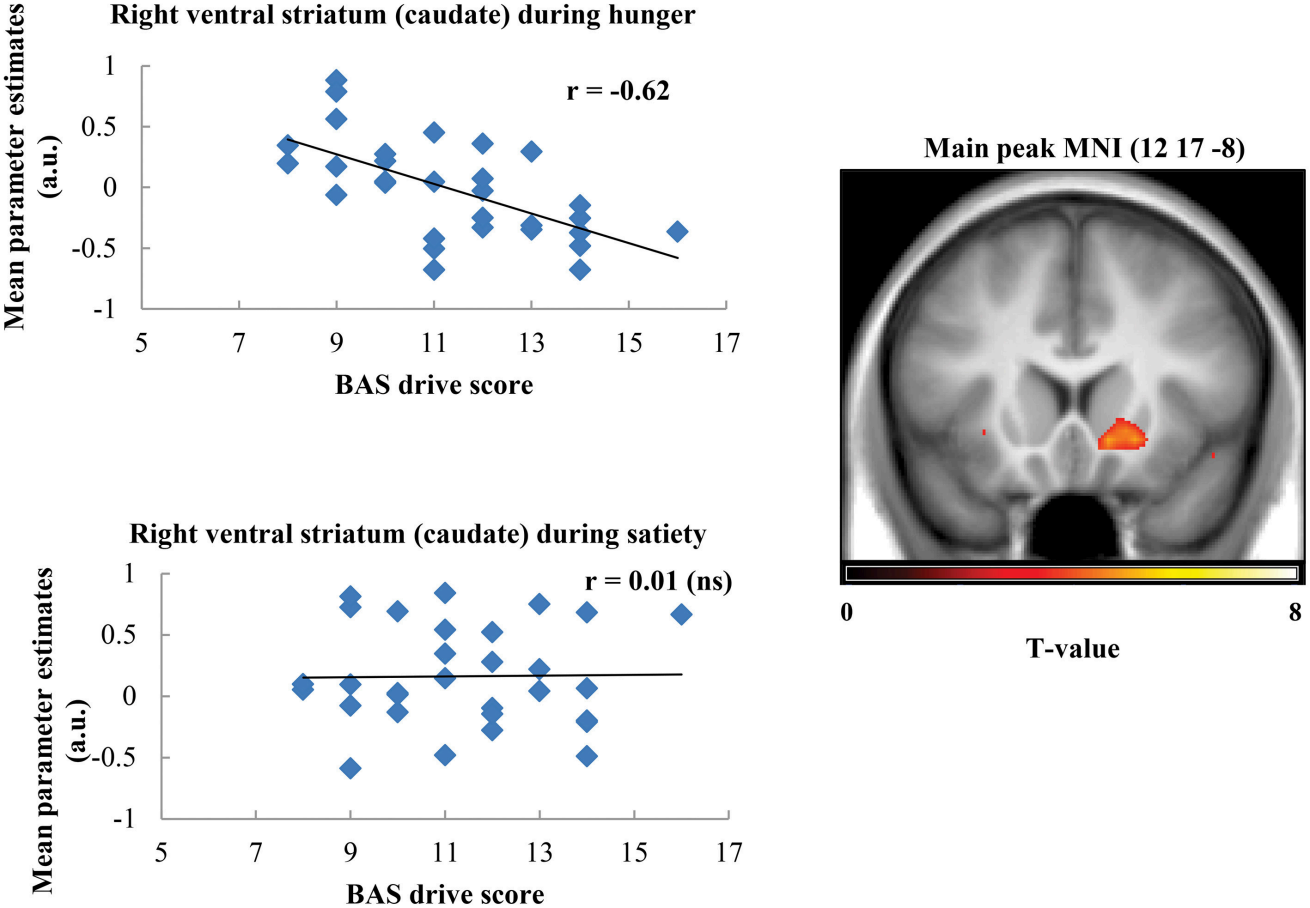
**Scatterplot of brain activation in response to oral calories (maltodextrin and sucralose minus sucralose) and reward sensitivity (BAS drive score) during hunger (significant) and satiety (not significant) in the right ventral striatum (caudate)**.

**Figure 2:**
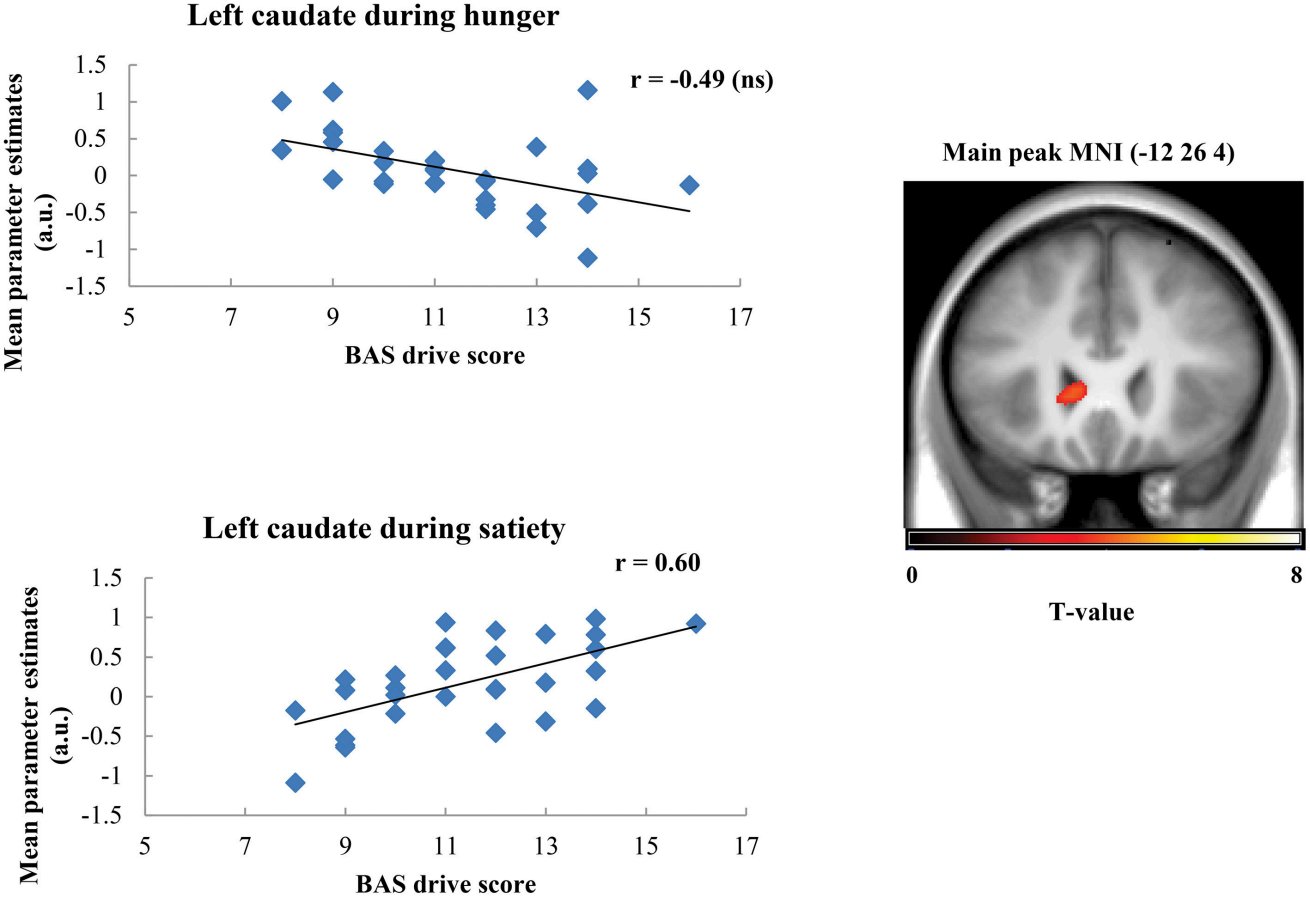
**Scatterplot of brain activation in response to oral calories (maltodextrin and sucralose minus sucralose) and reward sensitivity (BAS drive score) during hunger (not significant) and satiety (significant) in the left caudate**.

**Figure 3:**
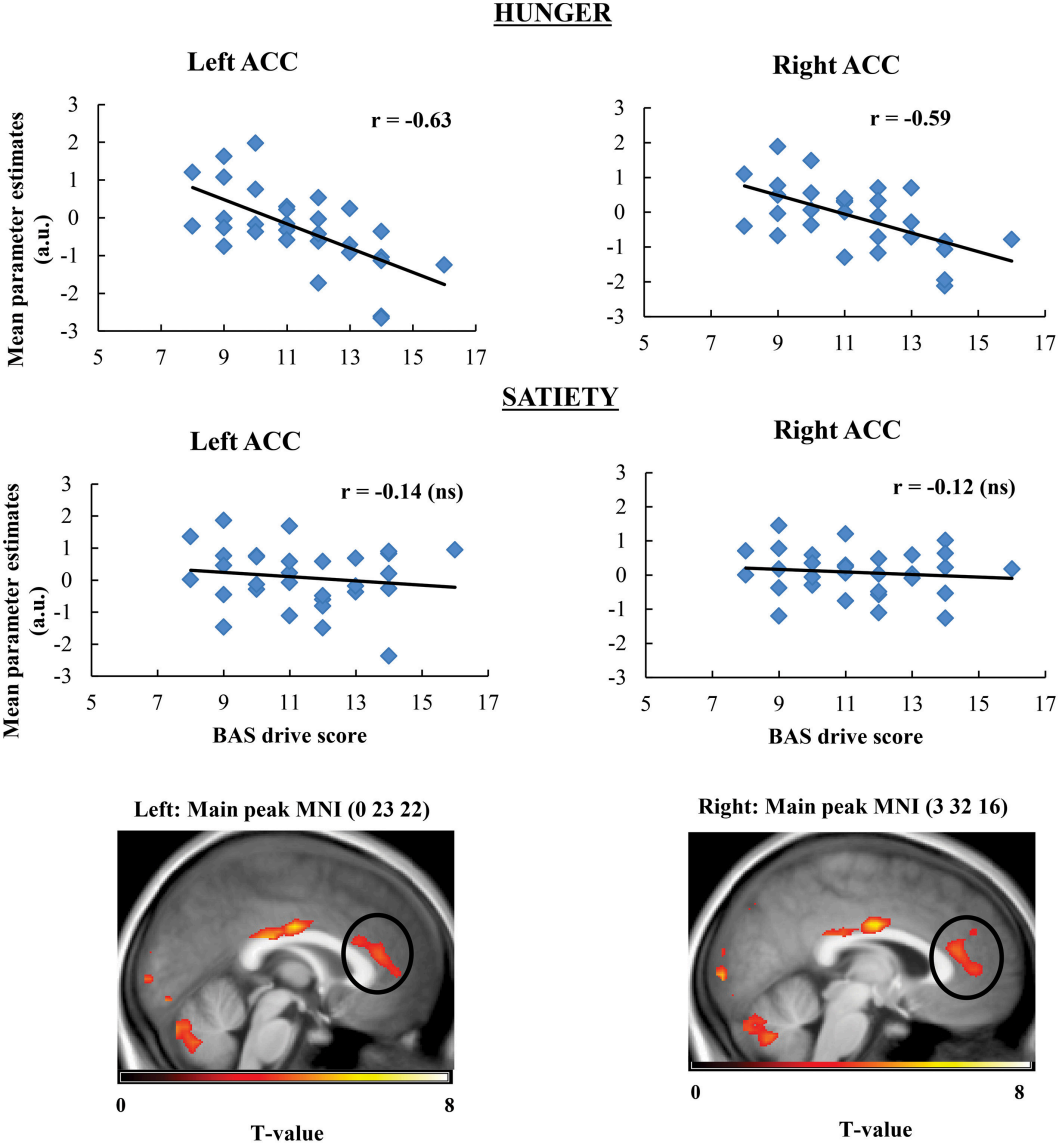
**Scatterplot of brain activation in response to oral calories (maltodextrin and sucralose minus sucralose) and reward sensitivity (BAS drive score) during hunger (significant) and satiety (not significant) in the left and right ACC**.

**Figure 4:**
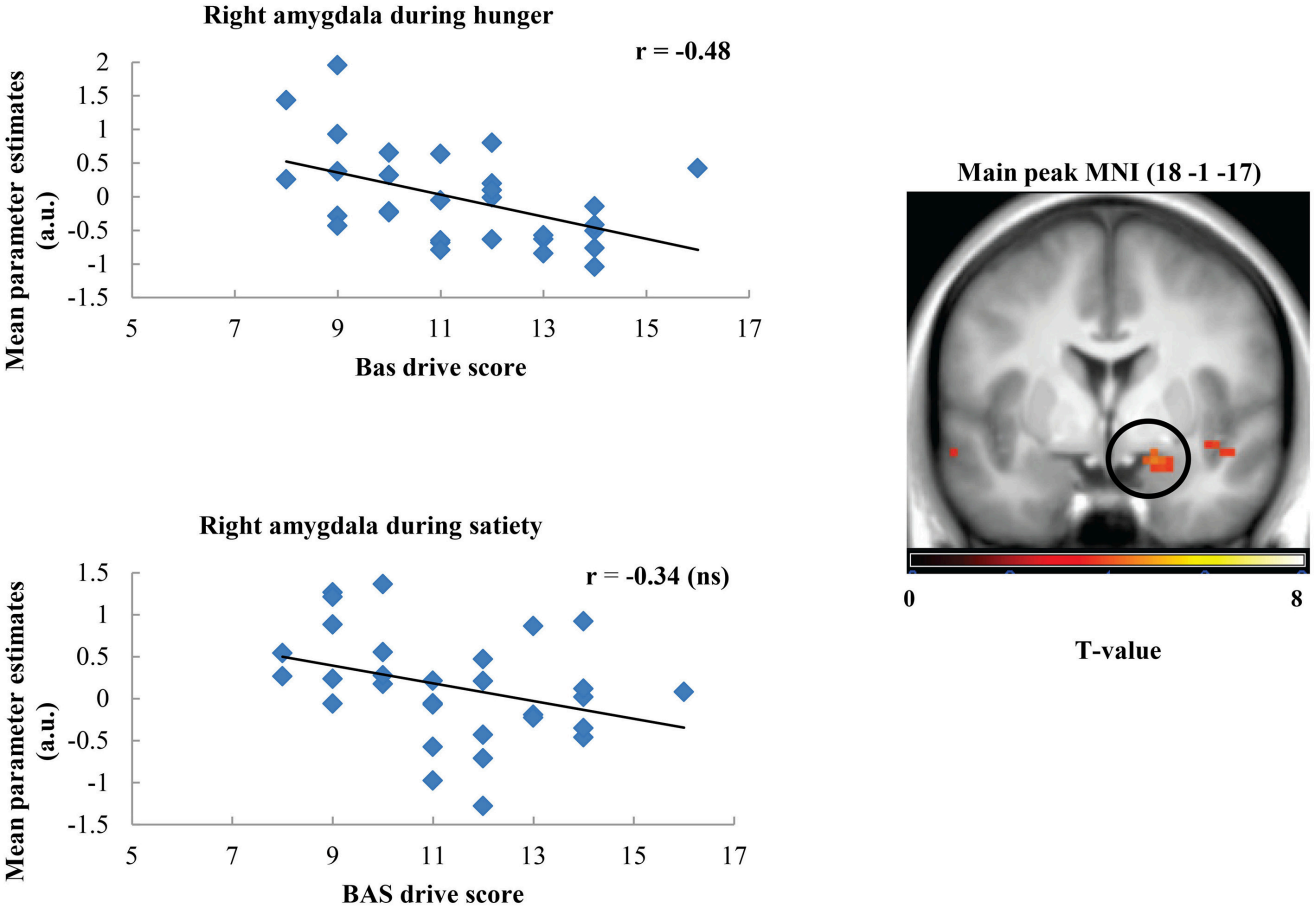
**Scatterplot of brain activation in response to oral calories (maltodextrin and sucralose minus sucralose) and reward sensitivity (BAS drive score) during hunger (significant) and satiety (not significant) in the right amygdala**.

Taste activation in the right caudate (ventral striatum) correlated negatively with BAS drive scores during hunger (*r* = −0.62; Figure 1). During satiety, however, BAS drive scores were positively correlated with activation in the left caudate (*r* = 0.60; Figure 2). Taste activation in the ACC (bilaterally) and the right amygdala correlated negatively with BAS drive scores during hunger (left ACC: *r* = −0.63, right ACC: *r* = −0.59, right amygdala: *r* = −0.48), but not during satiety (Figures 3, 4).

### Study 2: soft drink and reward sensitivity

Brain activation during tasting of soft drinks with vs. without calories did not correlate with BAS drive and BAS reward scores in any of the ROIs.

## Discussion

We assessed the correlation between reward sensitivity and brain responses to calories in the mouth in different hunger states. Firstly, we assessed this in data from a functional neuroimaging study, in which simple solutions of a non-caloric sweetener with or without maltodextrin were administered during hunger and satiety (van Rijn et al., 2015). We found that when participants were hungry and tasted calories, brain activation in the right ventral striatum (caudate), amygdala and ACC (bilaterally) correlated negatively with BAS drive scores. In contrast, when participants were satiated, brain responses correlated positively with BAS drive scores in the left caudate. BAS reward scores did not correlated with taste activation in reward related areas.

Secondly, we sought to extrapolate these findings to regular drinks by assessing the relationship between brain responses to calories in the mouth and reward sensitivity in data from a study in which soft drinks sweetened with either sucrose or a non-caloric sweetener were administered during hunger (Griffioen-Roose et al., 2013). Here, we found no correlations between reward sensitivity and brain responses to calories in any reward related area.

For simple solutions, correlations with taste activation were found for BAS drive but not for BAS reward. The lack of findings for BAS reward may be explained by the valence of the solutions. BAS reward is related to the degree of positive feelings people experience in response to a reward. Solutions were, on average, disliked by the participants (mean liking scores on 9-point scale: 2.9 (maltodextrin + sucralose), 3.4 (sucralose), see van Rijn et al., 2015) and probably did not elicit positive feelings. BAS drive scores, which are related to the tendency to take action in response to a food reward, did correlate with the response to calories in the brain reward system. Thus, the response to oral calories is associated with BAS drive, independent of stimulus valence.

Brain responses during food-viewing have been found to correlate with reward sensitivity in the caudate, amygdala and ACC (Beaver et al., 2006; Schienle et al., 2009). We focused on brain responses during exposure to another food-cue: the presence of calories in the oral cavity. In line with the food-viewing studies, we found that taste activation in the striatum, amygdala and ACC is correlated with reward sensitivity. Both the striatum and ACC are important in encoding food reward. They were found to be consistently activated in a meta-analysis of 28 studies in response to a pleasant tastant (Sescousse et al., 2013). The amygdala has also been implicated in food reward (Smeets et al., 2006; Grabenhorst et al., 2010; Jacobson et al., 2010) In addition, several other studies found that the striatum, ACC and amygdala are also involved in the neural encoding of oral calories (Frank et al., 2008; Smeets et al., 2011; Griffioen-Roose et al., 2013) Our results extend this by showing that activation in response to oral calories in the ACC, caudate and amygdala varies with the degree to which individuals are sensitive to reward.

We found an inverse relationship between reward sensitivity and the brain response to oral calories in the amygdala, ACC and caudate. The amygdala plays a central role in the emotional processing of sensory stimuli (Zald, 2003; Costafreda et al., 2008; Sergerie et al., 2008). Aversive stimuli have been found to activate the amygdala (Zald et al., 1998; O'Doherty et al., 2001). However, positive stimuli may also deactivate it (Zald, 2003). This might explain our inverse relationship in the amygdala, because calories can been seen as positive stimuli. In line with this, previous research also showed that tasting a caloric soft drink deactivates the amygdala (Smeets et al., 2011).

Concerning the caudate, Smeets et al. (2011) showed an opposite effect, namely that tasting a caloric soft drink resulted in more activation than tasting a non-caloric one. Few studies with a fMRI-taste paradigm have reported deactivation in the striatum. At this moment, it is known that omission of an expected reward can produce deactivations in the ventral striatum (McClure et al., 2003; O'Doherty, 2004). However, in the current study there was no negative prediction error, thus this cannot explain the negative correlation in the striatum. We speculate that an alternative explanation for caudate deactivation might be the firing of GABA-neurons. The basal ganglia exert inhibitory control over several motor areas via GABAergic output (Hikosaka, 2007). The presence of calories in the mouth compared to a non-caloric liquid, might induce such firing to inhibit motor movements such as searching for other foods. GABA-neurons of individuals with a higher reward-sensitivity level might respond stronger to calories.

Another explanation for the deactivation in the striatum, ACC and amygdala might be that the response to calories in individuals with lower sensitivity to reward is more adapted to internal hunger. If so, they may experience calories as more rewarding during hunger, when calories are necessary for survival, and as less rewarding during satiety, when calories are not necessary. In line with this explanation, we found a positive correlation between reward sensitivity and caudate activation in response to oral calories during satiety.

For soft drinks, we found no correlations with reward sensitivity in any reward related area during hunger. The discrepancy between this finding and the associations found for simple solutions can be explained in a number of ways. Firstly, the source of calories was different: maltodextrin vs. sucrose. Sucrose activates the sweet taste receptor, i.e., calories from sucrose are signaled by sweetness. On the contrary, maltodextrin, a tasteless substance for humans (Sclafani and Mann, 1987), is most likely directly detected by an oral maltodextrin receptor, independent of sweet taste (Lapis et al., 2014). In line with this, brain activation is different for a simple sugar compared to maltodextrin (Chambers et al., 2009). Thus, the different calorie sources may trigger different signaling mechanisms, which could have led to different results. In addition, it must be noted that we did not explicitly test for and excluded maltodextrin-tasters in the study with simple solutions. Previous research showed that a small percentage of the population can taste maltodextrin (de Araujo et al., 2013). This could have amplified the results. Secondly, one study used unfamiliar solutions whereas the other study used familiar products (soft drinks that were very similar to commercially available variants). In both studies, we only included participants that consumed more sugar sweetened than artificially sweetened beverages in daily life. Therefore, we assume that participants were conditioned to link the flavor of the soft drinks to calories (Appleton et al., 2006; Brunstrom and Mitchell, 2007; O'sullivan et al., 2010). This conditioning might have overruled the effect of actual caloric content. Thirdly, the studies used participants of different genders. This may have led to dissimilar results because male and female brain responses to food can differ (Smeets et al., 2006; Uher et al., 2006; Cornier et al., 2010; Frank et al., 2010; Haase et al., 2011). In particular, women may respond stronger to external food-related stimuli than men (Uher et al., 2006; Frank et al., 2010), which could explain why we find effects for women, but not men. Finally, the study with mainly men included fewer participants (*n* = 18). Many fMRI papers with a tasting-paradigm have used a comparable sample size and have shown significant results, for example: Bender et al. (2009) (*n* = 19), Frank et al. (2008) (*n* = 12), Haase et al. (2009) (*n* = 18), O'Doherty et al. (2001) (*n* = 7), Spetter et al. (2010) (*n* = 15). Nevertheless, it is possible that the relatively low sample size has prevented detection of small effects.

## Conclusion

We found that neural responses to oral calories from a maltodextrin solution are modulated by reward sensitivity in reward-related areas such as the caudate, amygdala, and ACC. This was not the case for a sucrose sweetened soft drink. This discrepancy may be due to the direct detection of maltodextrin, but not sucrose in the oral cavity. Also, in a familiar drink (soft drink), detection of calories *per se* may be overruled by a conditioned response to the familiar flavor. In conclusion, the brain reward response to calories from a long chain starch sugar (maltodextrin) varies with reward sensitivity. The absence of this effect in a familiar soft drink warrants further research into its relevance for real life ingestive behavior.

## Author contributions

IV, SG, CD, PS: Substantial contributions to the conception or design of the work; or the acquisition, analysis, or interpretation of data for the work; IV, SG, CD, PS: Drafting the work or revising it critically for important intellectual content; IV, SG, CD, PS: Final approval of the version to be published; IV, SG, CD, PS: Agreement to be accountable for all aspects of the work in ensuring that questions related to the accuracy or integrity of any part of the work are appropriately investigated and resolved.

## Funding

This work is part of the FOCOM project which was supported by the European Regional Development Fund and the Dutch Provinces Gelderland and Overijssel (Grant number 2011-017004).

### Conflict of interest statement

The authors declare that the research was conducted in the absence of any commercial or financial relationships that could be construed as a potential conflict of interest.
