# Supplementary material for: Neural Processing of Calories in Brain Reward Areas Can be Modulated by Reward Sensitivity
Source: Front Behav Neurosci. 2016 Jan 14;9:371. doi: 10.3389/fnbeh.2015.00371 (PMC4712268; doi:10.3389/fnbeh.2015.00371)
Supplement: Supplementary file 1 [file Table1.DOCX]

Supplementary Table 1. Brain regions in which brain activation by oral calories (maltodextrin and sucralose minus sucralose) correlated significantly with reward sensitivity (BAS drive score) during hunger and satiety.

|  |  |  |  |  |  |  |
| --- | --- | --- | --- | --- | --- | --- |
|  |  |  |  | Peak coordinates | | |
| ***Contrast*** | ***Region*** | ***Cluster size*** | ***Z-score*** | **x** | **y** | **z** |
|  |  |  |  |  |  |  |
|  |  |  |  |  |  |  |
| **Hunger** |  |  |  |  |  |  |
| *Positive correlation* | No regions were found |  |  |  |  |  |
| *Negative correlation* | R median cingulate | 66 | 4.79 | 3 | -10 | 31 |
|  | R posterior cingulate |  | 3.46 | 6 | -40 | 22 |
|  | L cerebellum | 409 | 4.51 | -33 | -58 | -26 |
|  | L calcarine sulcus |  | 3.92 | -3 | -85 | -11 |
|  | Vermis |  | 3.83 | 3 | -67 | -35 |
|  | L fusiform gyrus |  | 3.64 | -27 | -82 | -17 |
|  | R cerebellum |  | 3.24 | 6 | -70 | -29 |
|  | R cerebellum | 102 | 4.42 | 24 | -67 | -26 |
|  | L superior frontal gyrus | 169 | 4.39 | -18 | 47 | -2 |
|  | L middle frontal gyrus |  | 3.76 | -36 | 53 | 10 |
|  | L superior medial frontal gyrus |  | 3.37 | -15 | 65 | 4 |
|  | L thalamus | 27 | 4.35 | -12 | -4 | -2 |
|  | L calcarine sulcus | 13 | 4.29 | 3 | -94 | 1 |
|  | R caudate | 55 | 4.15 | 12 | 17 | -8 |
|  | R putamen |  | 4.09 | 21 | 17 | -8 |
|  | R rectus |  | 3.80 | 12 | 23 | -11 |
|  | R amygdala |  | 3.44 | 18 | 11 | -14 |
|  | R middle frontal gyrus | 37 | 3.91 | 24 | 41 | 4 |
|  | R superior frontal gyrus |  | 3.22 | 15 | 56 | 4 |
|  | R amygdala | 21 | 3.85 | 18 | -1 | -17 |
|  | R inferior temporal gyrus | 34 | 3.83 | 48 | -52 | -11 |
|  | R anterior cingulate | 80 | 3.72 | 3 | 32 | 16 |
|  | R medial frontal gyrus (orb) |  | 3.36 | 9 | 41 | -5 |
|  | L anterior cingulate |  | 3.33 | 0 | 23 | 22 |
|  | L precuneus | 14 | 3.44 | -9 | -70 | 37 |
|  | L superior parietal gyrus |  | 3.14 | -12 | -73 | 43 |
|  | R hippocampus | 14 | 3.35 | 42 | -10 | -17 |
|  | R insula |  | 3.27 | 42 | -1 | -11 |
|  | R middle frontal gyrus (orb) | 13 | 3.21 | 39 | 50 | -2 |
|  | R middle frontal gyrus |  | 3.20 | 39 | 53 | 7 |
|  |  |  |  |  |  |  |
| **Satiety** |  |  |  |  |  |  |
| *Positive correlation* | L caudate | 20 | 3.76 | -12 | 26 | 4 |
| *Negative correlation* | R cerebellum | 15 | 3.81 | 27 | -82 | -38 |
|  | L mid temporal gyrus | 10 | 3.62 | -51 | -34 | 10 |
|  |  |  |  |  |  |  |
|  |  |  |  |  |  |  |
