# Supplementary material for: Neural Processing of Calories in Brain Reward Areas Can be Modulated by Reward Sensitivity
Source: Front Behav Neurosci. 2016 Jan 14;9:371. doi: 10.3389/fnbeh.2015.00371 (PMC4712268; doi:10.3389/fnbeh.2015.00371)
Supplement: Supplementary file 2 [file Table2.DOCX]

Supplementary Table 2. Brain regions in which brain activation by oral calories (maltodextrin and sucralose minus sucralose) correlated significantly with reward sensitivity (BAS reward score) during hunger and satiety.

|  |  |  |  |  |  |  |
| --- | --- | --- | --- | --- | --- | --- |
|  |  |  |  | Peak coordinates | | |
| ***Contrast*** | ***Region*** | ***Cluster size*** | ***Z-score*** | **x** | **y** | **z** |
|  |  |  |  |  |  |  |
|  |  |  |  |  |  |  |
| **Hunger** |  |  |  |  |  |  |
| *Positive correlation* | R cerebellum | 21 | 3.93 | 9 | -73 | -29 |
| *Negative correlation* | L lingual gyrus | 25 | 3.61 | -27 | -61 | -2 |
|  |  |  |  |  |  |  |
| **Satiety** |  |  |  |  |  |  |
| *Positive correlation* | No regions were found |  |  |  |  |  |
| *Negative correlation* | R inferior frontal gyrus (tri) | 22 | 4.08 | 45 | 35 | 1 |
|  | R insula | 19 | 3.86 | 39 | 5 | 1 |
|  |  |  |  |  |  |  |
|  |  |  |  |  |  |  |
