# Supplementary material for: Neural Processing of Calories in Brain Reward Areas Can be Modulated by Reward Sensitivity
Source: Front Behav Neurosci. 2016 Jan 14;9:371. doi: 10.3389/fnbeh.2015.00371 (PMC4712268; doi:10.3389/fnbeh.2015.00371)
Supplement: Supplementary file 3 [file Table3.DOCX]

Supplementary Table 3. Brain regions in which brain activation by oral calories (sugar sweetened soft drink minus non-caloric sweetened soft drink) correlated significantly with reward sensitivity (BAS reward score) during hunger.

|  |  |  |  |  |  |  |
| --- | --- | --- | --- | --- | --- | --- |
|  |  |  |  | Peak coordinates | | |
| ***Contrast*** | ***Region*** | ***Cluster size*** | ***Z-score*** | **x** | **y** | **z** |
|  |  |  |  |  |  |  |
|  |  |  |  |  |  |  |
| **Hunger** |  |  |  |  |  |  |
| *Negative correlation* | L Precentral gyrus | 20 | 3.87 | -42 | 2 | 58 |
|  |  |  |  |  |  |  |
|  |  |  |  |  |  |  |
